# A hybrid cost-sensitive ensemble for heart disease prediction

**DOI:** 10.1186/s12911-021-01436-7

**Published:** 2021-02-25

**Authors:** Qi Zhenya, Zuoru Zhang

**Affiliations:** 1 College of Management and Economics, Tianjin University, Nankai District, Tianjin, 300072 People’s Republic of China; 2 School of Mathematical Science, Hebei Normal University, Yuhua District, Shijiazhuang, 050024 People’s Republic of China

**Keywords:** Cost-sensitive, Ensemble, Heart disease

## Abstract

**Background:**

Heart disease is the primary cause of morbidity and mortality in the world. It includes numerous problems and symptoms. The diagnosis of heart disease is difficult because there are too many factors to analyze. What’s more, the misclassification cost could be very high.

**Methods:**

A cost-sensitive ensemble method was proposed to improve the efficiency of diagnosis and reduce the misclassification cost. The proposed method contains five heterogeneous classifiers: random forest, logistic regression, support vector machine, extreme learning machine and k-nearest neighbor. T-test was used to investigate if the performance of the ensemble was better than individual classifiers and the contribution of Relief algorithm.

**Results:**

The best performance was achieved by the proposed method according to ten-fold cross validation. The statistical tests demonstrated that the performance of the proposed ensemble was significantly superior to individual classifiers, and the efficiency of classification was distinctively improved by Relief algorithm.

**Conclusions:**

The proposed ensemble gained significantly better results compared with individual classifiers and previous studies, which implies that it can be used as a promising alternative tool in medical decision making for heart disease diagnosis.

## Background

Heart disease is any disorder that influences the heart’s ability to function normally [1]. As the leading cause of death, heart disease is responsible for nearly $30\%$ of the global deaths annually [2]. In China, it is estimated that 290 millon people are suffering from heart disease, and the rate of death caused by heart disease is more than $40\%$ [3]. According to The European Society of Cardiology (ESC), nearly half of the heart disease patients die within initial 2 years [4]. Therefore, accurate diagnosis of heart disease in early stages is of great importance in improving security of heart [5].

However, as it’s associated with numerous symptoms and various pathologic features such as diabetes, smoking and high blood pressure, the diagnosis of heart disease remains a huge problem for less experienced physicians [6]. In order to detect heart disease, several diagnostic methods have been developed, Coronary angiography (CA) and Electrocardiography (ECG) are the most widely used among them, but they both have serious defects. ECG may fail to detect the symptoms of heart disease in its record [7] while CA is invasive, costly and needs highly-trained operators [8].

Computer-aided diagnostic methods based on machine learning predictive models can be noninvasive if they are based on the data that can be gathered using noninvasive methods, they can also help physicians make proper and objective diagnoses, hence reduce the suffering of patients [9]. Various machine learning predictive models [10–14] have been developed and widely used for decision support in diagnosing heart disease. Dogan et al. [15] built a random forest (RF) classification model for coronary heart disease. The clinical characteristics of the 1545 and 142 subjects were used for training and testing respectively, and the classification accuracy of symptomatic coronary heart disease was $78\%$. Detrano et al. [16] proposed a logistic regression (LR) classifier for heart disease classification and obtained an accuracy of $77\%$ in 3 patient test groups. Gokulnath and Shantharajah [17] proposed a classification model based on genetic algorithm (GA) and support vector machine (SVM), obtaining an accuracy of $88.34\%$ on Cleveland heart disease dataset. Subbulakshmi et al. [18] performed a detailed analysis of different activation functions of extreme learning machine (ELM) using Statlog heart disease dataset. The results indicated that ELM achieved an accuracy of $87.5\%$, higher than other methods. Duch et al. [19] used K-nearest neighbor (KNN) classifier to predict heart disease on Cleveland heart disease dataset and achieved an accuracy of $85.6\%$, superior to other machine learning techniques.

As No Free Lunch Theorem implies, no single model or algorithm can solve all classification problems [20]. One way to overcome the limitations of a single classifier is to use an ensemble model. An ensemble model is the combination of multiple sets of classifiers, it can outperform the individual classifiers because the variance of error estimation is reduced [21–24]. In recent years, many ensemble approaches have been proposed to improve the performance of heart disease diagnosis systems. For instance, Das et al. [25] proposed a neural networks ensemble and obtained $89.01\%$ classification accuracy from the experiments made on the data taken from Cleveland heart disease dataset. Bashir et al. [26] employed the ensemble of five heterogeneous classifiers on five heart disease datasets. The proposed ensemble classifier achieved the high diagnosis accuracy of $87.37\%$. Khened et al. [27] presented an ensemble system based on deep fully convolutional neural network (FCNN) and achieved a maximum classification accuracy of $100\%$ on Automated Cardiac Diagnosis Challenge (ACDC-2017) dataset. Therefore, we use an ensemble classifier to predict the presence or absence of heart disease in present study.

From the previous studies, it is observed that traditional medical decision support systems usually focused only on the maximization of classification accuracy without taking the unequal misclassification costs between different categories into consideration. However, in the field of medical decision making, it is often the minority class that is of higher importance [28]. Further, the cost associated with missing a patient (false negative) is much higher than that of mislabeling a healthy instance (false positive) [29]. Therefore, traditional classifiers inevitably result in a defective decision support system. In order to overcome this limitation, in this paper we combine the classification results of individual classifiers in a cost-sensitive way so that classifiers that help reduce the costs gain more weights in the final decision.

The rest of the paper is organized as follows. Section "Data-mining algorithms" offers brief background information concerning Relief algorithm and each individual classifier. Section "Methods" presents the framework of the proposed cost-sensitive ensemble. Section "Experimental setup" illustrates the research design of this paper in detail. Section "Results" describes the experimental results and compares the ensemble method with individual classifiers and previous methods. In section "Discussion", experimental results are discussed in detail. Finally, the conclusions and directions for future works are summarized in section "Conclusions".

## Data-mining algorithms

### Relief feature selection algorithm

Relief is a kind of famous filter feature selection algorithm which adopts a relevant statistics to measure the importance of the feature. This statistics can be seen as the weight of each feature. Top *k* features of bigger weights are selected. Therefore, the key is to determine the relevant statistics [30].

Assume $D = \{(x_1, y_1), (x_2, y_2), \ldots (x_m, y_m)\}$ is a dataset. $x_i$ is an input feature vector and $y_i$ is a class label corresponding to $x_i$. First, select a sample $x_i$ randomly. Then, Relief attempts to find out its nearest sample $x_{i,nh}$ from samples of its same class and nearest sample $x_{i,nm}$ from samples of its different class using the same techniques as in KNN, $x_{i,nh}$ is called “near-hit”, $x_{i,nm}$ is called “near-miss”. Next, update the weight of a feature *A* in *W* as described in Algorithm 1 [31, 32]. Repeat the random sampling steps for *m* times and get the average value of *W*[*A*], *W*[*A*] is the weight of feature *A*.

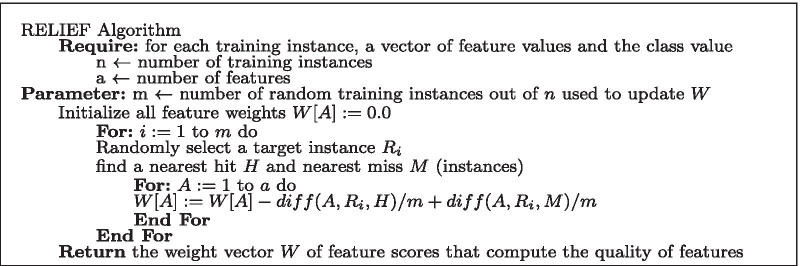



In Algorithm 1, $diff(x_{a}^j, x_{b}^j)$ depends on the type of feature *j*. For discrete feature *j*:

$$\begin{aligned} diff(x_{a}^j, x_{b}^j) = \left\{ \begin{aligned} 0,&x_{a}^j = x_{b}^j\\ 1,&otherwise, \end{aligned} \right. \end{aligned}$$

for continuous feature *j*:

$$\begin{aligned} diff(x_{a}^j, x_{b}^j) = | x_{a}^j - x_{b}^j |. \end{aligned}$$

Repeatedly operate for *n* times, then average the weights of each feature. Finally, choose the top *k* features for classification.

### Machine learning classifiers

Machine learning classification algorithms are used to distinguish heart disease patients from healthy people. Five popular classifiers and their theoretical backgrounds are discussed briefly in this paper.

#### Random forest

RF is a machine learning algorithm based on the ensemble of decision trees [33]. In traditional decision tree methods such as C4.5 and C5.0, all the features are used for generating the decision tree. In contrast, RF builds multiple decision trees and chooses the random subspaces of the features for each of them. Then, the votes of trees are aggregated and the class with the most votes is the prediction result [34]. As an excellent classification model, RF can successfully reduce the overfitting and calculate the nonlinear and interactive effects of variables. Besides, the training of each tree are done separately, so it could be done in parallel, which reduced the training time needed. Finally, combining the prediction result of each tree could reduce the variance and improve the accuracy of the predictions. There are many studies showing the performance superiority of RF over other machine learning methods [35–37].

#### Logistic regression

LR is a generalized linear regression model [38]. Therefore, it is similar with multiple linear regression in many aspects. Usually, LR is used for binary classification problems where the predictive variable $y \in [0,1]$, 0 is negative class and 1 is positive class. But it can also be used for multi-classification.

In order to distinguish heart disease patients from healthy people, a hypothesis $h(\theta ) = \theta ^TX$ is proposed. The threshold of classifier output is $h_{\theta }(x) = 0.5$, which is to say, if the value of hypothesis $h_{\theta }(x) \ge 0.5$, it will predict $y = 1$ which means that the person is a heart disease patient, otherwise the person is healthy. Hence, the prediction is done.

The sigmoid function of LR can be written as:

$$\begin{aligned} h_{\theta }(x) = \frac{1}{1+e^{-z}}, \end{aligned}$$

where $$z = \theta ^TX$$.

The cost function of LR can be written as:

$$\begin{aligned} J(\theta ) = \frac{1}{m}\sum _{i=1}^mcost ( y_i, y_i' ), \end{aligned}$$

where *m* is the number of instances to be predicted, $y_i$ is the real class label of the *i*th instance, and $y_i'$ is the predicted class label of the *i*th instance.

$$\begin{aligned} cost ( y_i, y_i' ) = \left\{ \begin{aligned} 0,&\quad y_i = y_i'\\ 1,&\quad otherwise. \end{aligned} \right. \end{aligned}$$

#### Support vector machine

Invented by Cortes and Vapnik [39], SVM is a supervised machine learning algorithm which has been widely used for classification problems [29, 40, 41]. The output of SVM is in the form of two classes in a binary classification problem, making it a non-probabilistic binary classifier [42]. SVM tries to find a linear maximum margin hyperplane that separates the instances.

Assume the hyperplane is $w^Tx+b=0$, where *w* is a dimensional coefficient vector, which is normal to the hyperplane of the surface, *b* is offset value from the origin, and *x* is dataset values. Obviously, the hyperplane is determined by *w* and *b*. The data points nearest to the hyperplane are called support vectors. In the linear case, *w* can be solved by introducing Lagrangian multiplier $\alpha _i$. The solution of *w* can be written as:

$$\begin{aligned} w = \sum _{i=1}^m\alpha _iy_ix_i, \end{aligned}$$

where *m* is the number of support vectors and $y_i$ are target labels to *x*. The linear discriminant function can be written as:

$$\begin{aligned} g(x)=sgn\left(\sum _{i=1}^m\alpha _iy_ix_i^Tx+b\right), \end{aligned}$$

*sgn* is the sign function that calculates the sign of a number, $sgn(x)=-1$ if $x< 0$, $sgn(x)=0$ if $x=0$, $sgn(x)=1$ if $x> 0$. The nonlinear separation of data set is performed by using a kernel function. The discriminant function can be written as:

$$g(x)=sgn\left(\sum_{i=1}^m\alpha_iy_iK(x_i,x)+b\right),$$

where $K(x_i,x)$ is the kernel function.

#### Extreme learning machine

ELM was first proposed by Huang et al. [43]. Similar to a single layer feed-forward neural network(SLFNN), ELM is also a simple neural network with a single hidden layer. However, unlike a traditional SLFNN, the hidden layer weights and bias of ELM are randomized and need not to tune, and the output layer weights of ELM are analytically determined through simple generalized inverse operations [43, 44].

#### K-nearest neighbor

KNN a supervised classification algorithm. Its procedure is as follows: when a new case is given, first search the database to find the *k* historical cases which are closest to the new case, namely k-nearest neighbors, and then these neighbors vote on the class label of the new case. If a class has the most nearest neighbors, the new case is determined to belong to the class [45]. The following formula is used to calculate the distance between two cases [46]:

$$\begin{aligned} d(x_i,x_j)=\sum _{q\in Q}w_q(x_{iq}-x_{jq})^2+\sum _{c\in C}w_cL_c(x_{ic},x_{jc}), \end{aligned}$$

where *Q* is the set of quantitative features and *C* is the set of categorical features, $L_c$ is an $M \times M$ symmetric matrix, $w_q$ is the weight of feature *q* and $w_c$ is the weight of feature *c*.

## Methods

**Fig. 1:**
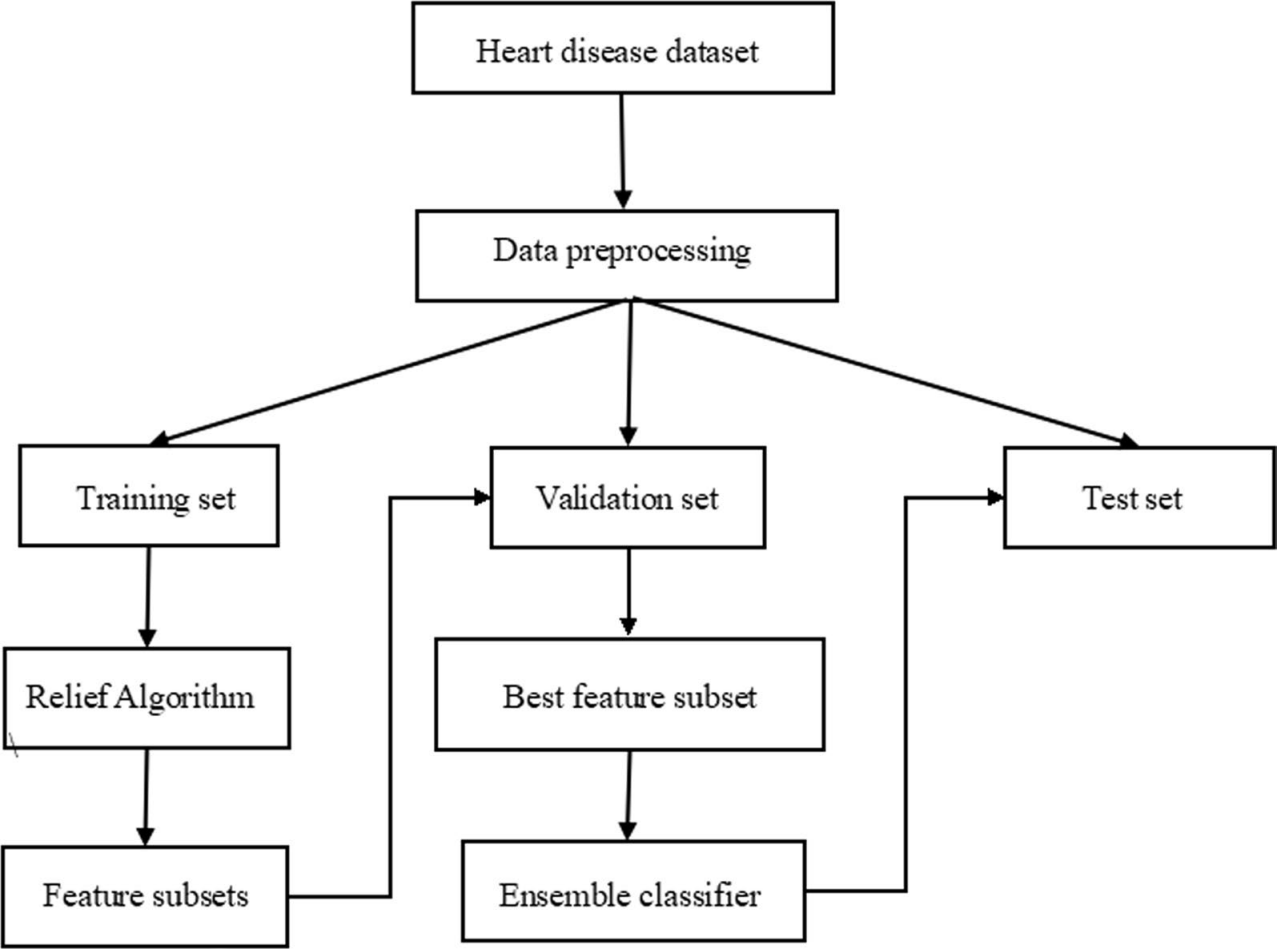
Flowchart of the proposed ensemble classifier

The proposed classification system consists of four main components: (1) preprocessing of data, (2) feature selection using Relief algorithm, (3) training of individual classifiers, and (4) prediction result generation of the ensemble classifier. A flow chart of the proposed system is shown in Fig. 1. The main components of the system are described in the following subsections.

### Data preprocessing

The aim of data preprocessing is to obtain data from different heart disease data repositories and then process them in the appropriate format for the subsequent analysis [47]. The preprocessing phase involves missing-value imputation and data normalization.

#### Missing-value imputation

Missing data in medical data sets must be handled carefully because they have a serious effect on the experimental results. Usually, researchers choose to replace the missing values with the mean/mode of the attribute depending on its type [26]. Mokeddem [47] used weighted KNN to calculate the missing values. In present study, features with missing values more than $50\%$ of all instances are removed, then group mean instead of simple mean are used to substitute remaining missing values, as Bashir et al did in their study [41]. For example, if the case with a missing value is a patient, the mean value for patients is calculated and inserted in place of the missing value. In this way the class label is taken into consideration, thus the information offered by the dataset could be fully utilized.

#### Data normalization

Before feature selection, the continuous features are normalized to ensure that they have the mean 0 and variance 1, thus the effects of different quantitative units are eliminated.

### Feature selection and training of individual classifiers

In this phase, the dataset is randomly split into training set, validation set and test set. That is, $80\%$ of the dataset is used for training, $10\%$ is used for validation and $10\%$ is used for testing purpose. The features are selected by the Relief algorithm on training set and the obtained result is a feature rank. A higher ranking means that the feature has stronger distinguishing quality and a higher weight [48]. Afterwards, features are added to the ensemble model one by one, from the most important one to the least. Then we can get several models with different number of features using training set, the number of models equals to the number of features. These models are tested on validation set, and the ensemble classifier with the best performance should have the best feature subset. Such classifier is used on test set, and its performance is recorded in Sect. 5. This procedure is repeated 10 times.

### Prediction result generation

The classification accuracy and misclassification cost (MC) of each classifier are taken into account during the process of generating the final prediction result. In present study, in order to compare the misclassification costs for the different classifiers conveniently, the value of the correct classification cost is set as 0, and the MC is split into two scenarios. In the first scenario, healthy people are diagnosed with heart disease, resulting in unnecessary and costly treatment. In the second scenario, heart disease patients are told that they are healthy, as a result they may miss the best time for treatment, which may cause the disease to deteriorate or even death. The cost matrix is presented in Table 1. Considering the different costs people have to pay for misclassification, we set $cost_1=10$ and $cost_2=1$ [49, 50]. Afterwards, an index *E* is constructed to evaluate the performance of each classifier:

$$\begin{aligned} E_i= \frac{Accuracy_i+1-\frac{MC_i}{cost_1+cost_2}}{2}, \end{aligned}$$

where $Accuracy_i$ represents the accuracy and $MC_i$ represents the MC of *i*th classifier during the training phase (the formula to calculate the MC is presented in Sect. 4.2). $E_i$ stands for the efficiency of *i*th classifier to improve the accuracy and reduce the MC simultaneously. The weights of individual classifiers are based on $E_i$ and they are calculated as:

$$\begin{aligned} w_i=\frac{E_i}{\sum \limits _{i=1}^nE_i}, \end{aligned}$$

where *n* is the number of classifiers. Finally, the instances of the test set are imported into each classifier, and the outputs of ensemble classifier are the labels with the highest weighted vote [51].

**Table 1:** The cost matrix used by the classifiers

Predicted	Reality	
	Patients	Controls
Patients	0	$cost_2$
Controls	$cost_1$	0

**Table 2:** Number of patients in each dataset

Dataset	Patients	Controls	% Patients	% Controls
Statlog	120	150	44.44	55.56
Cleveland	139	164	45.87	54.13
Hungarian	106	188	36.05	63.95

## Experimental setup

In this section, details of datasets are discussed. The detail of evaluation metrics and their significance is presented as well. The experiment is implemented on MATLAB 2018a platform, and the performance parameters of the executing host were Win 10, Inter (R) 1.80 GHz Core (TM) i5-8250U, X64, and 16 GB (RAM). In present study, the number of decision trees to build the RF is 50, the Gaussian kernel function is used in SVM, and the number of k is 5 in KNN. The parameters of individual classifiers are chosen by genetic algorithm. The fitness function is the E value of the proposed ensemble classifier. The population size is set to be 50. The crossover fraction is 0.8. The migration fraction is 0.2. The generations are 1000.

### Datasets description

Three different datasets are used in the proposed research, they are Statlog, Cleveland and Hungarian heart disease datasets from UCI machine learning repository [52]. Statlog dataset consists of 270 instances, Cleveland dataset consists of 303 instances and Hungarian dataset consists of 294 instances. The number of heart disease patients in each dataset is presented in Table 2. The three datasets share the same feature set. Details of feature information are presented in Table 3.

**Table 3:** Features of heart disease datasets

Feature	Description	Value
Age	Age in years	Continuous value
Sex	Sex	1: male; 0: female
Cp	Chest pain type	1: typical angina
		2: atypical angina
		3: non-anginal pain
		4: asymptomatic
Trestbps	Resting blood sugar	Continuous value in mm hg
Chol	Serum Cholestoral	Continuous value in mm/dl
Fbs	Fasting blood sugar	$0: <120$ mg/dl
		$1: >120$ mg/dl
Restecg	Resting ECG results	0 : normal
		1 : having ST-T wave abnormality
		2 : probable or definite
		left ventricular hypertrophy
Thalach	Maximum heart rate achieved	Continuous value
Exang	Exercise induced angina	0 : no
		1 : yes
Oldpeak	ST depression induced by exercise relative to rest	Continuous value
Slope	Slope of the peak exercise ST segment	1 = upsloping
		2 = flat
		3 = downsloping
Ca	Number of major vessels colored by flourosopy	0, 1, 2, 3
Thal	Heart beat	3 : normal
		6 : fixed defect
		7 : reversable defect
Num	Predicted class	0, 1

**Table 4:** Feature ranking on different datasets

Feature	Statlog	Cleveland	Hungarian
Age	9	9	7
Sex	4	4	2
Cp	1	1	1
Trestbps	8	8	5
Chol	13	13	6
Fbs	11	12	10
Restecg	7	7	8
Thalach	12	10	9
Exang	6	5	4
Oldpeak	10	11	3
Slope	5	6	$\backslash$*
Ca	2	2	$\backslash$*
Thal	3	3	$\backslash$*

*Means that feature is deleted during data preprocessing

### Performance evaluation metrics

Various performance metrics are used to evaluate the performance of the classifiers in this study. In the confusion matrix, the classification result of a two-class problem is divided into four parts: true positive (TP), true negative (TN), false positive (FP) and false negative (FN). Based on these error measures, E, MC, G-mean, precision, specificity, recall and AUC are used to evaluate the performance of different classifiers. As accuracy is included in the calculation of E, it is not used as an evaluation metric alone. The metrics are calculated as follows:

1 $$\begin{aligned} MC&= {} \frac{FP\times cost_2+FN\times cost_1}{TP+TN+FP+FN} \times 100\%, \end{aligned}$$

2 $$\begin{aligned} G-mean&= {} \sqrt{\frac{TP}{TP+FN}\times \frac{TN}{TN+FP}} \times 100\%, \end{aligned}$$

3 $$\begin{aligned} Precision&= {} \frac{TP}{TP+FP} \times 100\%, \end{aligned}$$

4 $$\begin{aligned} Specificity&= {} \frac{TN}{TN+FP} \times 100\%, \end{aligned}$$

5 $$\begin{aligned} Recall&= {} \frac{TP}{TP+FN}\times 100\%. \end{aligned}$$

Ten-fold cross validation is used to obtain the final results. The ensemble classifier runs on each test set and processes each instance individually. The evaluation metrics of the ten folds are averaged to verify the superiority of the proposed ensemble classifier. Wilcoxon signed-rank test is used on all three datasets to examine if the new method is statistically better than single classifiers and check if the contribution of the Relief algorithm is significant.

## Results

This section involves the exhibition of experimental results on different heart disease datasets.

### Feature ranking on different datasets

**Fig. 2:**
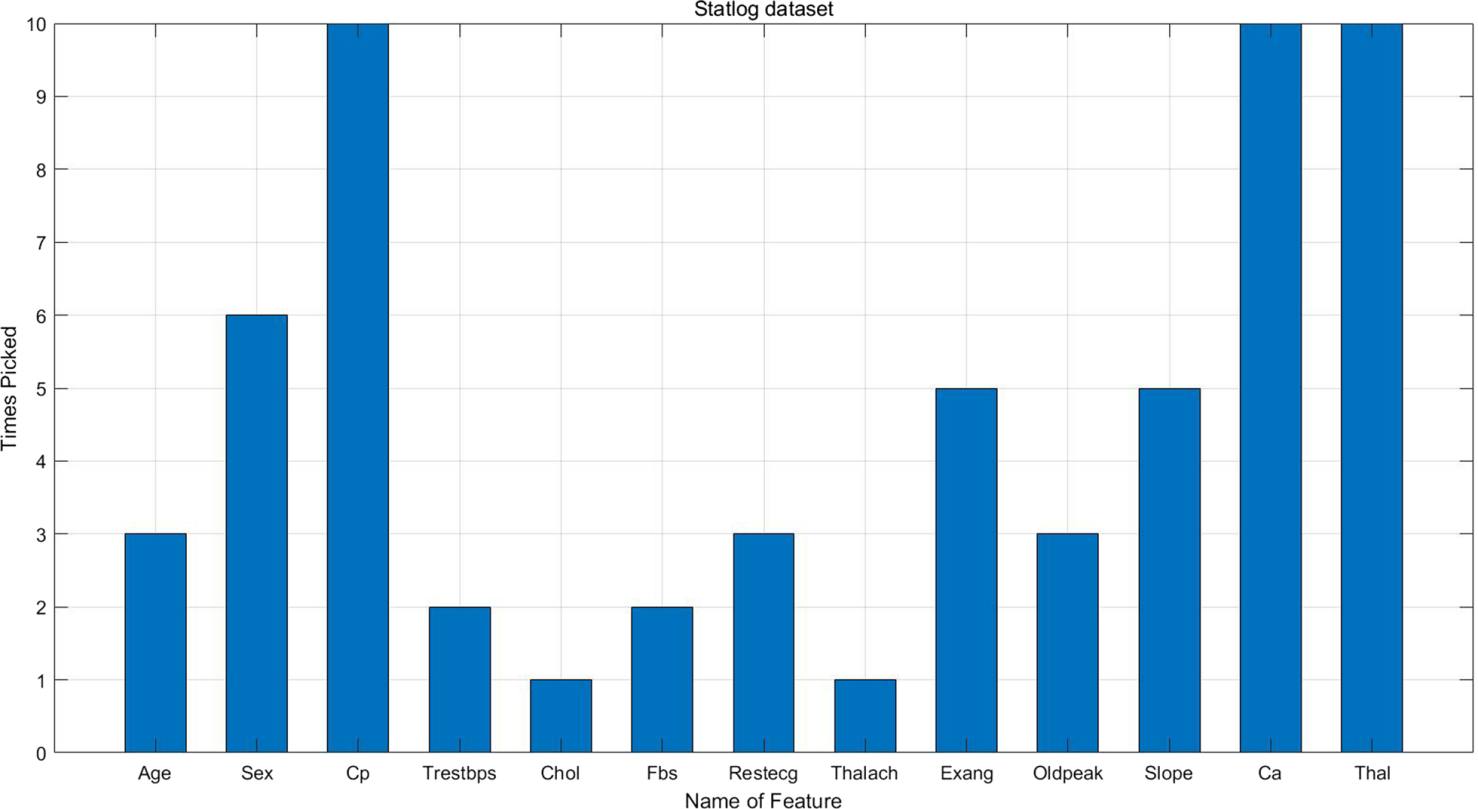
Times picked for each feature on Statlog datasets

**Fig. 3:**
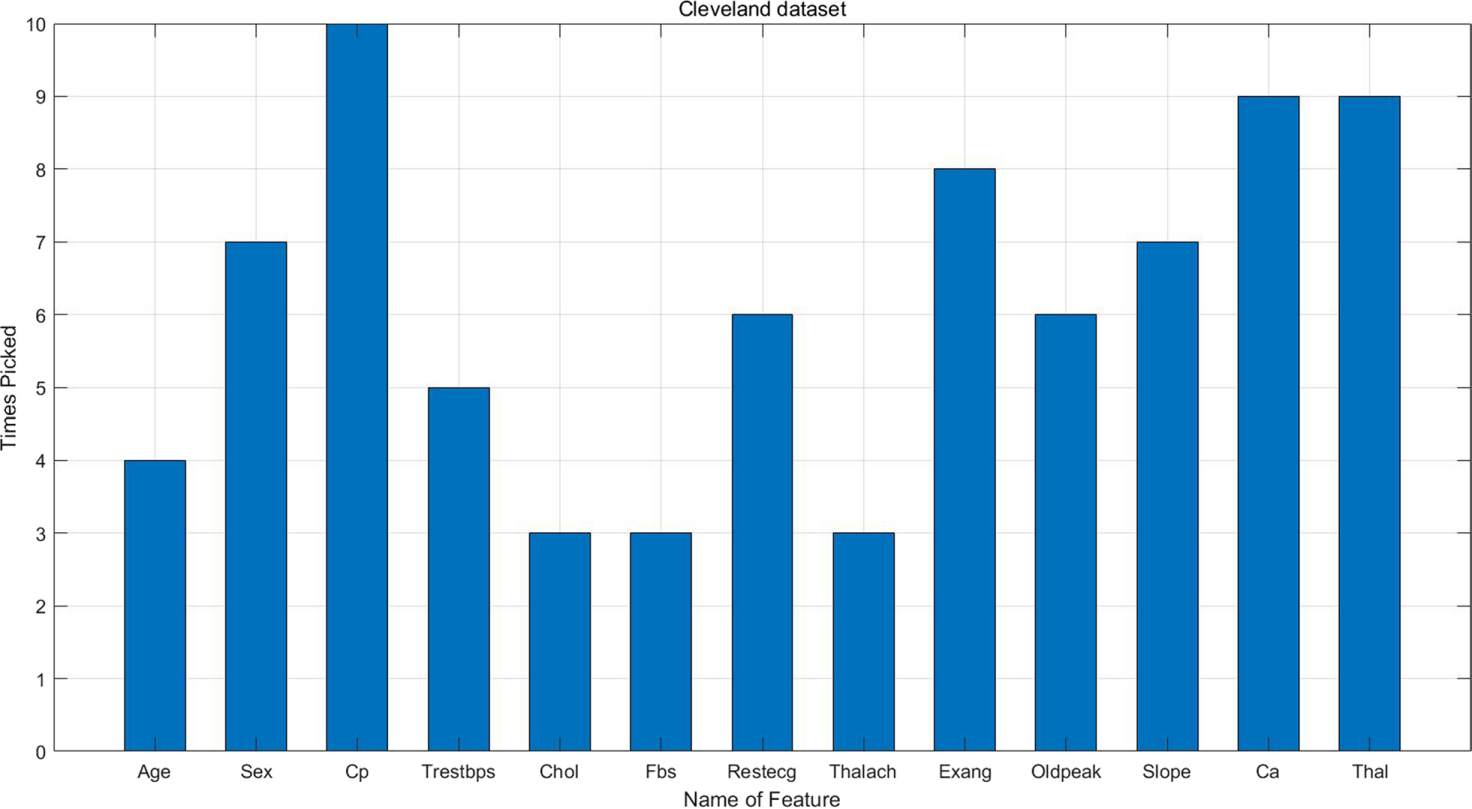
Times picked for each feature on Cleveland datasets

**Fig. 4:**
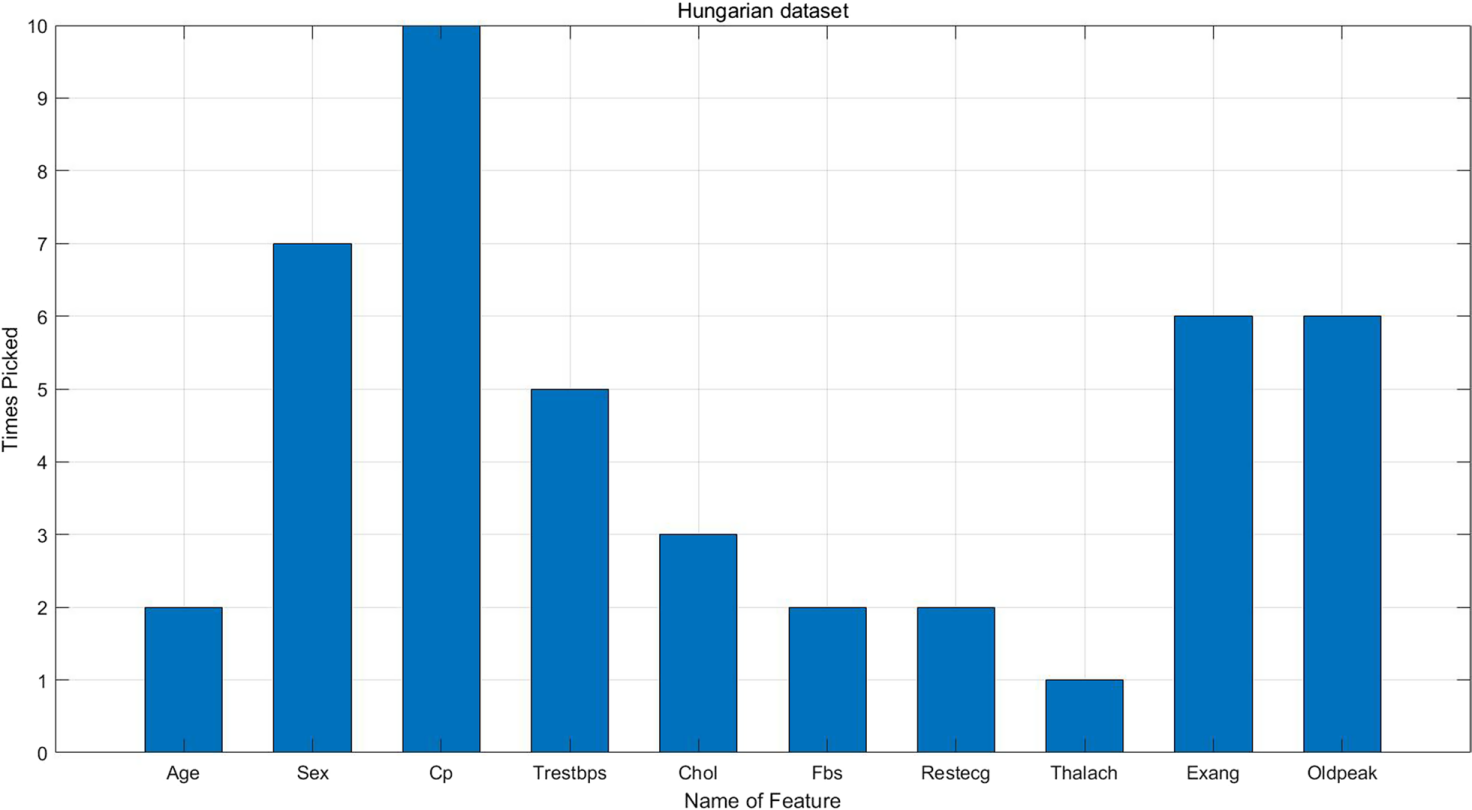
Times picked for each feature on Hungarian datasets

Table 4 shows feature ranking on the three heart disease datasets. For Hungarian dataset, Slope, Ca and Thal are deleted during the process of missing-value imputation because these features have missing values more than $50\%$ of all instances. Therefore, only ten features are ranked. Figures 2, 3 and 4 illustrate how many times a certain feature is chosen to enter the best feature subset in the whole experiment. As we can see, sex, Cp, Exang, Slope, Ca and Thal are the most important features on Statlog dataset; sex, Cp, Restecg, Exang, Oldpeak, Slope, Ca and Thal are the most important features Cleveland dataset; sex, Cp, Trestbps, Exang and Oldpeak are the most important features on Hungarian dataset.

**Table 5:** Experimental results on Statlog dataset with the best feature subset

Mean ± SD	RF	LR	SVM	ELM	KNN	Proposed ensemble
E (%)	87.53 ± 5.39	87.87 ± 6.82	88.67 ± 5.02	82.81 ± 5.54	76.94 ± 11.33	**94.44 ± 3.78**
Precision (%)	83.70 ± 6.58	84.07 ± 8.01	84.81 ± 6.40	78.15 ± 6.64	70 ± 15.37	**92.59 ± 4.62**
Recall (%)	80.64 ± 11.80	82.08 ± 13.07	83.85 ± 10.98	70.65 ± 13.77	62.85 ± 17.51	**92.15 ± 7.10**
G-mean	83.14 ± 7.54	83.79 ± 8.19	84.41 ± 7.10	76.65 ± 8.05	68.40 ± 15.63	**92.56 ± 4.79**
MC (%)	51.85 ± 26.07	50 ± 34.67	44.81 ± 23.78	75.19 ± 29.63	96.67 ± 44.56	**22.22 ± 19.36**
Specificity (%)	86.13 ± 6.17	86 ± 6.58	85.45 ± 7.83	84.29 ± 8.43	75.18 ± 16.55	**93.21 ± 5.43**
AUC (%)	83.75 ± 8.26	83.92 ± 9.44	85.07 ± 7.72	80.17 ± 6.96	68.42 ± 13.73	**92.08 ± 5.51**

The average $+-$ sd on 10-folds CV. The best result is bolded

### Performance on Statlog dataset

Table 5 indicates the comparison of performance evaluation metrics for the proposed ensemble with individual classifiers on Statlog dataset. It is clear from the results that the proposed ensemble algorithm has obtained the highest E of $94.44\pm 3.78\%$, the highest precision of $92.59\pm 4.62\%$, the highest recall of $92.15\pm 7.10\%$, the highest G-mean of $92.56\pm 4.79\%$, the highest specificity of $93.21\pm 5.43\%$, the highest AUC of $92.08\pm 5.51\%$ and the lowest MC of $22.22\pm 19.36\%$. SVM is ranked second at the E level achieving $88.67\pm 5.02\%$. The result of Wilcoxon signed-rank test comparing the proposed ensemble and individual classifiers is shown in Table 6. It can be seen that the performance of proposed ensemble is significantly superior to individual classifiers on most of the metrics, except specificity with RF.

**Table 6:** Wilcoxon signed-rank test: proposed ensemble versus individual classifiers on Statlog dataset

	RF	LR	SVM	ELM	KNN
E					
Z-value$^{1}$	− 2.805	− 2.805	− 2.670	− 2.670	− 2.803
*p* value	$$0.002^{**}$$	$$0.002^{**}$$	$$0.004^{**}$$	$$0.004^{**}$$	$$0.002^{**}$$
Precision					
Z-value	− 2.692	− 2.829	− 2.680	− 2.677	− 2.807
*p* value	$$0.004^{**}$$	$$0.002^{**}$$	$$0.004^{**}$$	$$0.004^{**}$$	$$0.002^{**}$$
Recall					
Z-value	− 2.374	− 2.527	− 2.388	− 2.670	− 2.536
*p* value	$$0.016^{*}$$	$$0.008^{**}$$	$$0.016^{*}$$	$$0.004^{**}$$	$$0.008^{**}$$
G-mean					
Z-value	− 2.803	− 2.803	− 2.666	− 2.666	− 2.803
*p* value	$$0.002^{**}$$	$$0.002^{**}$$	$$0.004^{**}$$	$$0.004^{**}$$	$$0.002^{**}$$
MC					
Z-value	− 2.654	− 2.805	− 2.670	− 2.670	− 2.803
*p* value	$$0.006^{**}$$	$$0.002^{**}$$	$$0.004^{**}$$	$$0.004^{**}$$	$$0.002^{**}$$
Specificity					
Z-value	− 1.825	− 2.243	− 2.371	− 2.673	− 2.675
*p* value	0.086	$$0.023^{*}$$	$$0.016^{*}$$	$$0.004^{**}$$	$$0.004^{**}$$
AUC					
Z-value	− 2.547	− 2.599	− 2.668	− 2.666	− 2.666
*p* value	$$0.008^{**}$$	$$0.006^{**}$$	$$0.004^{**}$$	$$0.004^{**}$$	$$0.004^{**}$$

$^{{1}}$The value of Wilcoxon statistics after standardization

* $$p < 0.05$$** $$p < 0.01$$

In order to investigate the contribution of Relief algorithm, experiments are done on Statlog dataset with all the features to make a comparison. The result is shown in Table 7. The proposed ensemble algorithm has obtained the highest E of $86.36\pm 5.51\%$, the highest precision of $78.52\pm 7.37\%$, the highest recall of $92.56\pm 8.19\%$, the highest G-mean of $90.17\pm 8.08\%$, the highest specificity of $87.84\pm 5.73\%$, the highest AUC of $87.99\pm 8.39\%$ and the lowest MC of $34.81\pm 24.58\%$. ELM is ranked second at the E level achieving $77.31\pm 8.11\%$. Compared with Table 5, the ensemble classifier with all the features is worse than that with feature subset chosen by Relief algorithm. Table 8 gives the result of Wilcoxon signed-rank test between the two algorithms, from which we can reach the conclusion that the difference is significant. In addition, it can be seen from Fig. 2 that only 6 features on average are chosen by Relief algorithm for prediction, which reduces the computation largely.

**Table 7:** Experimental results on Statlog dataset with 13 features

Mean ± SD	RF	LR	SVM	ELM	KNN	Proposed ensemble
E (%)	71.76 ± 7.44	77.16 ± 4.53	68.49 ± 6.06	77.31 ± 8.11	66.70 ± 4.26	**86.36** ± **5.51**$^*$
Precision (%)	65.19 ± 14.96	73.70 ± 7.77	68.15 ± 10.03	61.48 ± 29.28	59.26 ± 11.05	**78.52** ± **7.37**
Recall (%)	86.54 ± 10.48	83.13 ± 8.57	75.62 ± 6.28	82.45 ± 18.42	73.57 ± 13.65	**92.56** ± **8.19**
G-mean	82.18 ± 9.64	83.72 ± 14.18	76.29 ± 7.45	82.60 ± 14.51	76.35 ± 18.16	**90.17** ± **8.08**
MC (%)	75.12 ± 9.10	56.30 ± 7.77	62.69 ± 25.27	41.12 ± 33.75	85.19 ± 43.82	**34.81** ± **24.58**
Specificity (%)	78.05 ± 7.26	84.32 ± 8.97	76.96 ± 16.40	82.81 ± 8.72	79.23 ± 17.11	**87.84** ± **5.73**
AUC (%)	79.35 ± 11.28	83.16 ± 9.78	83.16 ± 9.82	81.27 ± 12.51	78.53 ± 6.94	**87.99** ± **8.39**

$*$ The average $+-$ sd on 10-folds CV. The best result is bolded.

### Performance on Cleveland dataset

Table 9 shows the classification result of each classifier with reduced feature subset. The proposed ensemble has achieved the highest E of $93.83\pm 4.93\%$, the highest precision of $88.67\pm 5.49\%$, the highest recall of $89.68\pm 8.78\%$, the highest G-mean of $90.77\pm 6.71\%$, the highest specificity of $89.31\pm 5.13\%$, the highest AUC of $89.54\pm 5.54\%$ and the lowest MC of $22.00\pm 15.61\%$. The ensemble classifier performs the best on all the evaluation metrics while KNN performs the worst. The result of Wilcoxon signed-rank test comparing the proposed ensemble and individual classifiers is shown in Table 10. The ensemble classifier is obviously better than other classifiers on different metrics except for specificity.

The performance of the proposed ensemble without Relief algorithm on Cleveland dataset is listed in Table 11. The proposed ensemble has achieved the highest E of $82.07\pm 6.00\%$, the highest precision of $83.79\pm 7.59\%$, the highest recall of $75.88\pm 11.08\%$, the highest G-mean of $79.76\pm 7.76\%$, the highest specificity of $84.16\pm 6.70\%$, the highest AUC of $79.53\pm 8.24\%$ and the lowest MC of $62.96\pm 26.52\%$. LR is ranked second at the E level achieving $77.29\pm 5.52\%$. It can be concluded that the ensemble classifier performs worse than that with reduced feature subset, which indicates that there are irrelevant and distractive features. Table 12 shows the Wilcoxon signed-rank test result between the two ensembles. As we can see, the classifiers gained significantly better performance with reduced feature subset. Besides, as shown in Fig. 3, Relief algorithm has cut down the number of features to 8 on average, simplifying the calculation.

**Table 8:** Wilcoxon signed-rank test: classifiers with feature subset versus classifiers with 13 features on Statlog dataset

	RF	LR	SVM	ELM	KNN	Ensemble
E						
Z-value$^{1}$	− 2.803	− 2.803	− 2.803	− 2.668	− 2.803	− 2.803
*p* value$$^{2}$$	$$0.002^{**}$$	$$0.002^{**}$$	$$0.002^{**}$$	$$0.004^{**}$$	$$0.002^{**}$$	$$0.002^{**}$$
Precision						
Z-value	− 2.803	− 2.395	− 2.803	− 2.395	− 2.803	− 2.803
*p* value	$$0.002^{**}$$	$$0.014^{*}$$	$$0.002^{**}$$	$$0.014^{*}$$	$$0.002^{**}$$	$$0.002^{**}$$
Recall						
Z-value	− 1.988	− 2.701	− 2.803	− 1.580	− 2.803	− 2.803
*p* value	$$0.027^{*}$$	$$0.004^{**}$$	$$0.002^{**}$$	0.131	$$0.002^{**}$$	$$0.002^{**}$$
G-mean						
Z-value	− 2.803	− 2.701	− 2.803	− 2.599	− 2.803	− 2.803
*p* value	$$0.002^{**}$$	$$0.004^{**}$$	$$0.002^{**}$$	$$0.006^{**}$$	$$0.002^{**}$$	$$0.002^{**}$$
MC						
Z-value	− 2.599	− 2.701	− 2.803	− 2.090	− 2.803	− 2.803
*p* value	$$0.006^{**}$$	$$0.004^{**}$$	$$0.002^{**}$$	$$0.037^{*}$$	$$0.002^{**}$$	$$0.002^{**}$$
Specificity						
Z-value	− 2.803	− 2.293	− 2.802	− 2.599	− 2.599	− 2.803
*p* value	$$0.002^{**}$$	$$0.020^{*}$$	$$0.002^{**}$$	$$0.006^{**}$$	$$0.006^{**}$$	$$0.002^{**}$$
AUC						
Z-value	− 2.803	− 2.701	− 2.803	− 2.803	− 2.803	− 2.803
*p* value	$$0.002^{**}$$	$$0.004^{**}$$	$$0.002^{**}$$	$$0.002^{**}$$	$$0.002^{**}$$	$$0.002^{**}$$

$^1$ The value of Wilcoxon statistics after standardization

$$^2$$ $$^*$$ $$p < 0.05$$, $$^{**}$$ $$p < 0.01$$

### Performance on Hungarian dataset

Figure 4 shows the times each feature is included in the best feature subset on Hungarian dataset. Table 13 indicates the experimental results on Hungarian dataset with feature subset chosen by Relief algorithm. The proposed ensemble classifier has achieved the highest E of $89.47\pm 3.06\%$, the highest precision of $89.31\pm 4.44\%$, the highest recall of $82.39\pm 5.73\%$, the highest G-mean of $82.95\pm 4.63\%$, the highest specificity of $92.02\pm 5.76\%$, the highest AUC of $88.38\pm 5.36\%$ and the lowest MC of $38.28\pm 12.10\%$. LR is ranked second at the E level achieving $82.07\pm 7.12\%$. The paired Wilcoxon signed-rank test between the ensemble and each classifier is listed in Table 14. The ensemble is significantly superior to other classifiers on most of the metrics except for specificity compared with RF,LR and SVM. This is because the proposed ensemble is cost-sensitive, one of its main aim is to identify patients as many as possible, thus the misclassification of healthy people is tolerable to a certain extent.

The performance of each classifier with all the features on Hungarian dataset is given in Table 15. The proposed ensemble classifier achieved the highest E of $79.87\pm 7.32\%$, the highest precision of $80.89\pm 7.89\%$, the highest recall of $66.38\pm 14.13\%$, the highest G-mean of $75.75\pm 9.22\%$, the highest specificity of $87.31\pm 3.60\%$, the highest AUC of $77.64\pm 8.31\%$ and the lowest MC of $74.08\pm 32.11\%$. Table 16 shows the Wilcoxon signed-rank test result between the ensemble with Relief algorithm and that without it. As we can see, the classifiers gained significantly better performance with reduced feature subset on most of the evaluation metrics.

**Table 9:** Experimental results on Cleveland dataset with the best feature subset

Mean ± SD	RF	LR	SVM	ELM	KNN	Proposed ensemble
E (%)	86.78 ± 6.15	86.53 ± 6.75	86.50 ± 5.89	84.19 ± 7.59	79.44 ± 9.05	**93.83** ± **4.93**$^{*}$
Precision (%)	82.67 ± 7.28	83.00 ± 7.45	82.00 ± 6.25	79.00 ± 8.32	72.00 ± 11.88	**88.67** ± **5.49**
Recall (%)	80.26 ± 14.28	78.02 ± 16.41	81.20 ± 15.12	77.86 ± 19.94	73.70 ± 14.34	**89.68** ± **8.78**
G-mean	82.24 ± 8.84	82.24 ± 9.12	81.51 ± 8.03	78.77 ± 11.80	72.01 ± 11.84	**90.77** ± **6.71**
MC (%)	54.67 ± 33.45	59.67 ± 38.12	54.00 ± 35.38	63.67 ± 42.95	78.67 ± 39.79	**22.00** ± **15.61**
Specificity (%)	84.63 ± 7.49	87.49 ± 6.38	82.47 ± 6.54	80.42 ± 7.43	71.26 ± 14.11	**89.31** ± **5.13**
AUC (%)	81.53 ± 8.75	81.99 ± 9.38	80.91 ± 8.14	79.99 ± 11.05	70.53 ± 12.65	** 89.54** ± **5.54**

*The average $+-$ SD on 10-folds CV. The best result is bolded

**Table 10:** Wilcoxon signed-rank test: proposed ensemble versus individual classifiers on Cleveland dataset

	RF	LR	SVM	ELM	KNN
E					
Z-value$^{1}$	− 2.668	− 2.312	− 2.655	− 2.810	− 2.803
*p* value$$^{2}$$	$$0.004^{**}$$	$$0.021^{*}$$	$$0.008^{**}$$	$$0.002^{**}$$	$$0.002^{**}$$
Precision					
Z-value	− 2.533	− 2.318	− 2.671	− 2.814	− 2.809
*p* value	$$0.011^{*}$$	$$0.016^{*}$$	$$0.006^{**}$$	$$0.002^{**}$$	$$0.002^{**}$$
Recall					
Z-value	− 2.668	− 2.173	− 2.524	− 2.668	− 2.668
*p* value	$$0.004^{**}$$	$$0.031^{*}$$	$$0.008^{**}$$	$$0.004^{**}$$	$$0.004^{**}$$
G-mean					
Z-value	− 2.666	− 2.310	− 2.703	− 2.803	− 2.803
*p* value	$$0.004^{**}$$	$$0.020^{*}$$	$$0.004^{**}$$	$$0.002^{**}$$	$$0.002^{**}$$
MC					
Z-value	− 2.668	− 2.312	− 2.655	− 2.810	− 2.803
*p* value	$$0.004^{**}$$	$$0.020^{*}$$	$$0.006^{**}$$	$$0.002^{**}$$	$$0.002^{**}$$
Specificity					
Z-value	− 1.892	− 1.696	− 2.316	− 2.521	− 2.553
*p* value	0.094	0.101	$$0.023^{*}$$	$$0.008^{**}$$	$$0.008^{**}$$
AUC					
Z-value	− 2.521	− 2.310	− 2.666	− 2.803	− 2.803
*p* value	$$0.008^{**}$$	$$0.020^{*}$$	$$0.004^{**}$$	$$0.002^{**}$$	$$0.002^{**}$$

$^{{1}}$ The value of Wilcoxon statistics after standardization

$$^{{2}}$$ $$^*$$ $$p < 0.05$$, $$^{**}$$ $$p < 0.01$$

**Table 11:** Experimental results on Cleveland dataset with 13 features

Mean ± SD	RF	LR	SVM	ELM	KNN	Proposed ensemble
E (%)	76.01 ± 5.39	77.29 ± 5.52	75.74 ± 6.15	68.29 ± 8.95	58.43 ± 4.32	**82.07 ± 6.00**$^{*}$
Precision (%)	74.23 ± 6.41	76.84 ± 5.14	75.16 ± 7.47	65.54 ± 11.57	50.26 ± 6.74	**83.79 ± 7.59**
Recall (%)	68.08 ± 7.92	69.40 ± 13.02	69.41 ± 12.68	56.75 ± 14.76	45.20 ± 7.59	**75.88 ± 11.08**
G-mean	71.05 ± 6.75	73.59 ± 6.58	71.61 ± 7.07	61.45 ± 12.32	49.71 ± 6.20	**79.76 ± 7.76**
MC (%)	87.19 ± 21.18	81.61 ± 27.83	82.08 ± 29.68	114.60 ± 37.19	152.39 ± 19.74	**62.96 ± 26.52**
Specificity (%)	74.50 ± 9.02	79.31 ± 9.11	74.80 ± 8.20	67.32 ± 11.32	49.20 ± 11.80	**84.16 ± 6.70**
AUC (%)	70.22 ± 7.74	72.18 ± 5.69	71.18 ± 7.73	66.75 ± 11.40	45.32 ± 8.33	** 79.53 ± 8.24**

The average $+-$ SD on 10-folds CV. The best result is bolded

**Table 12:** Wilcoxon signed-rank test: classifiers with feature subset versus Classifiers with 13 features on Cleveland dataset

	RF	LR	SVM	ELM	KNN	Ensemble
E						
Z-value$^{1}$	− 2.803	− 2.803	− 2.803	− 2.803	− 2.803	− 2.803
*p* value$$^{2}$$	$$0.002^{**}$$	$$0.002^{**}$$	$$0.002^{**}$$	$$0.002^{**}$$	$$0.002^{**}$$	$$0.002^{**}$$
Precision						
Z-value	− 2.599	− 2.701	− 2.497	− 2.293	− 2.803	− 2.803
*p* value	$$0.006^{**}$$	$$0.004^{**}$$	$$0.010^{*}$$	$$0.020^{*}$$	$$0.002^{**}$$	$$0.002^{**}$$
Recall						
Z-value	− 1.988	− 2.701	− 2.293	− 1.674	− 1.988	− 2.803
*p* value	$$0.049^{*}$$	$$0.004^{**}$$	$$0.020^{*}$$	0.132	$$0.049^{*}$$	$$0.002^{**}$$
G-mean						
Z-value	− 2.497	− 2.701	− 2.497	− 2.293	− 1.784	− 2.803
*p* value	$$0.010^{*}$$	$$0.004^{**}$$	$$0.010^{*}$$	$$0.020^{*}$$	0.084	$$0.002^{**}$$
MC						
Z-value	− 2.293	− 2.701	− 2.497	− 1.784	− 2.599	− 2.803
*p* value	$$0.020^{*}$$	$$0.004^{**}$$	$$0.010^{*}$$	0.084	$$0.006^{**}$$	$$0.002^{**}$$
Specificity						
Z-value	− 2.803	− 2.803	− 2.802	− 2.702	− 2.701	− 2.803
*p* value	$$0.002^{**}$$	$$0.002^{**}$$	$$0.002^{**}$$	$$0.004^{**}$$	$$0.004^{**}$$	$$0.002^{**}$$
AUC						
Z-value	− 2.701	− 2.701	− 2.803	− 2.090	− 2.803	− 2.803
*p* value	$$0.004^{**}$$	$$0.004^{**}$$	$$0.002^{**}$$	$$0.037^{*}$$	$$0.002^{**}$$	$$0.002^{**}$$

$^1$ The value of Wilcoxon statistics after standardization

$$^2$$ $$^*$$ $$p < 0.05$$, $$^{**}$$ $$p < 0.01$$

**Table 13:** Experimental results on Hungarian dataset with the best feature subset

Mean ± SD	RF	LR	SVM	ELM	KNN	Proposed ensemble
E (%)	80.43 ± 5.37	82.07 ± 7.12	78.91 ± 5.61	80.40 ± 6.86	75.43 ± 8.64	**89.47 ± 3.06**
Precision (%)	75.52 ± 5.96	77.93 ± 8.48	74.48 ± 6.54	75.86 ± 7.09	66.55 ± 14.99	**89.31 ± 4.44**
Recall (%)	60.19 ± 16.84	62.08 ± 15.89	53.38 ± 17.93	59.42 ± 19.49	61.36 ± 19.71	**82.39 ± 5.73**
G-mean	71.04 ± 8.34	73.72 ± 10.15	67.55 ± 9.21	70.97 ± 10.16	59.97 ± 24.07	**82.95 ± 4.63**
MC (%)	87.93 ± 34.95	82.76 ± 37.63	100.00 ± 36.09	90.34 ± 44.33	94.14 ± 30.89	**38.28 ± 12.10**
Specificity (%)	86.34 ± 9.83	88.99 ± 7.79	89.10 ± 11.61	88.13 ± 9.92	70.92 ± 25.22	**92.02 ± 5.76**
AUC (%)	74.07 ± 9.16	76.31 ± 10.87	71.96 ± 10.98	74.59 ± 9.55	69.07 ± 9.98	** 88.38 ± 5.36**

The average $+-$ sd on 10-folds CV. The best result is bolded

**Table 14:** Wilcoxon signed-rank test: proposed ensemble versus individual classifiers on Hungarian dataset

	RF	LR	SVM	ELM	KNN
E					
Z-value$^{1}$	− 2.312	− 2.244	− 2.668	− 2.821	− 2.803
*p* value$$^{2}$$	$$0.020^{*}$$	$$0.022^{*}$$	$$0.004^{**}$$	$$0.002^{**}$$	$$0.002^{**}$$
Precision					
Z-value	− 2.446	− 2.271	− 2.689	− 2.840	− 2.814
*p* value	$$0.016^{*}$$	$$0.023^{*}$$	$$0.004^{**}$$	$$0.002^{**}$$	$$0.002^{**}$$
Recall					
Z-value	− 2.075	− 2.100	− 2.670	− 2.814	− 2.310
*p* value	$$0.035^{*}$$	$$0.039^{*}$$	$$0.004^{**}$$	$$0.002^{**}$$	$$0.020^{*}$$
G-mean					
Z-value	− 2.429	− 2.293	− 2.668	− 2.805	− 2.803
*p* value	$$0.012^{*}$$	$$0.020^{*}$$	$$0.004^{**}$$	$$0.002^{**}$$	$$0.002^{**}$$
MC					
Z-value	− 1.956	− 2.041	− 2.668	− 2.821	− 2.803
*p* value	0.051	$$0.043^{*}$$	$$0.004^{**}$$	$$0.002^{**}$$	$$0.002^{**}$$
Specificity					
Z-value	− 1.955	− 1.960	− 1.365	− 2.668	− 2.803
*p* value	0.055	0.055	0.195	$$0.004^{**}$$	$$0.002^{**}$$
AUC					
Z-value	− 2.803	− 2.346	− 2.803	− 2.803	− 2.805
*p* value	$$0.002^{**}$$	$$0.016^{*}$$	$$0.002^{**}$$	$$0.002^{**}$$	$$0.002^{**}$$

$^1$ The value of Wilcoxon statistics after standardization

$$^2$$ $$^*$$ $$p < 0.05$$, $$^{**}$$ $$p < 0.01$$

### Comparison of the results with other studies

Tables 17, 18 and 19 showed the comparison of our model and previous methods. As class imbalance is widespread in medical datasets, accuracy itself is not a proper evaluation metric. Here, we use recall and specificity to make the comparison, which are used by these researches together. Recall is used to measure the percentage of distinguishing patients correctly, while specificity is used to measure the percentage of distinguishing healthy people correctly.

As we can see, on Statlog dataset, heuristic rough set has gained similar recall with the proposed model, and neural network ensemble has better performance on specificity compared with the proposed model. On Cleveland dataset, deep belief network and decision tree + fuzzy inference system perform better than the proposed ensemble. Beyond those methods, the proposed ensemble performs better than any other models. On Hungarian dataset, the present study has achieved the best performance, which implies that the proposed ensemble has certain strength in dealing with incomplete dataset.

The results state that our proposed method obtains superior and promising results in classifying heart disease patients. Taken recall and specificity together, the proposed ensemble classifier has better performance than most previous studies. In addition, most researchers did not take different kinds of misclassification costs into consideration, and the limitation is remedied in present study.

**Table 15:** Experimental results on Hungarian dataset with 10 features

Mean ± SD	RF	LR	SVM	ELM	KNN	Proposed ensemble
E (%)	72.73 ± 6.29	73.85 ± 7.06	72.72 ± 6.78	69.94 ± 8.26	60.09 ± 10.59	**79.87 ± 7.32**
Precision (%)	72.72 ± 8.17	73.38 ± 8.14	71.78 ± 8.31	69.18 ± 10.08	53.77 ± 13.27	**80.89 ± 7.89**
Recall (%)	49.00 ± 16.03	52.92 ± 14.85	44.30 ± 17.06	44.39 ± 20.61	37.77 ± 18.40	**66.38 ± 14.13**
G-mean	62.75 ± 11.18	65.96 ± 10.60	60.44 ± 12.46	58.39 ± 14.78	45.48 ± 14.87	**75.75 ± 9.22**
MC (%)	109.40 ± 31.01	103.24 ± 32.31	118.24 ± 33.24	123.00 ± 42.83	148.60 ± 48.07	**74.08 ± 32.11**
Specificity (%)	82.62 ± 5.75	83.40 ± 5.22	85.57 ± 5.62	80.65 ± 8.26	59.28 ± 13.55	**87.31 ± 3.60**
AUC (%)	67.38 ± 10.99	68.59 ± 10.98	65.43 ± 10.99	61.67 ± 13.98	50.81 ± 15.55	** 77.64 ± 8.31**

The average $+-$ sd on 10-folds CV. The best result is bolded

**Table 16:** Wilcoxon signed-rank test: Classifiers with feature subset versus Classifiers with 10 features on Hungarian dataset

	RF	LR	SVM	ELM	KNN	Ensemble
E						
Z-value$^{1}$	− 2.803	− 2.803	− 2.803	− 2.803	− 2.497	− 2.701
*p* value$$^{2}$$	$$0.002^{**}$$	$$0.002^{**}$$	$$0.002^{**}$$	$$0.002^{**}$$	$$0.010^{*}$$	$$0.004^{**}$$
Precision						
Z-value	− 2.293	− 2.497	− 1,478	− 2.497	− 1.886	− 2.701
*p* value	$$0.020^{*}$$	$$0.010^{*}$$	0.160	$$0.010^{*}$$	0.065	$$0.004^{**}$$
Recall						
Z-value	− 1.886	− 2.497	− 2.395	− 1.376	− 2.497	− 1.886
*p* value	0.065	$0.010^{*}$	$0.014^{*}$	0.193	$0.010^{*}$	0.065
G-mean						
Z-value	− 2.191	− 2.803	− 2.497	− 2.803	− 1.580	− 2.599
*p* value	$$0.027^{*}$$	$$0.002^{**}$$	$$0.010^{*}$$	$$0.002^{**}$$	0.131	$$0.006^{**}$$
MC						
Z-value	− 2.090	− 2.803	− 2.599	− 2.599	− 2.497	− 2.191
*p* value	$$0.037^{*}$$	$$0.002^{**}$$	$$0.006^{**}$$	$$0.006^{**}$$	$$0.010^{*}$$	$$0.027^{*}$$
Specificity						
Z-value	− 2.599	− 2.497	− 1.886	− 2.803	− 1.988	− 2.803
*p* value	$$0.006^{**}$$	$$0.010^{*}$$	0.065	$$0.002^{**}$$	$$0.049^{*}$$	$$0.002^{**}$$
AUC						
Z-value	− 2.599	− 2.803	− 2.803	− 2.701	− 2.701	− 2.803
*p* value	$$0.006^{**}$$	$$0.002^{**}$$	$$0.002^{**}$$	$$0.004^{**}$$	$$0.004^{**}$$	$$0.002^{**}$$

$^1$ The value of Wilcoxon statistics after standardization

$$^2$$ $$^*$$ $$p < 0.05$$, $$^{**}$$ $$p < 0.01$$

**Table 17:** Comparison of the proposed system outcome with previous researches for Statlog dataset

Author	Method	Recall (%)	Specificity (%)
Present study	Ensemble classifier	92.15	93.21
Marateb and Goudarzi [60]	Naive Bayes	78.51	88.74
Bashir et al. [41]	BagMOOV	73.47	91.01
Ceylan and Koyuncu [61]	PSO$^{*}$ neural network	80.83	89.33
Mokeddem and Ahmed [47]	Fuzzy classification model	89.17	84.00
Das et al. [25]	Neural network ensemble	80.95	95.91
Xiao et al. [62]	Heuristic Rough Set	92.33	87.50
Bashir et al. [26]	Ensemble model	87.50	87.27

$^{*}$ Particle swarm optimization

The values listed in the table represent the average performance on ten folds

**Table 18:** Comparison of the proposed system outcome with previous researches for Cleveland dataset

Author	Method	Recall (%)	Specificity (%)
Present study	Ensemble classifier	89.68	89.31
Kahramanli and Allahverdi [63]	Hybrid neural network	93	78.5
Shah et al. [64]	PPCA$^{1}$ + SVM	75	90.57
Marian and Filip [65]	Fuzzy rule-based classification	84.70	92.90
Ali et al. [56]	Gaussian Naive Bayes classifier	87.80	97.95
Ali et al. [57]	Deep neural network	85.36	100
Ali et al. [58]	Hybrid SVM	82.92	100
Ali et al. [59]	Deep belief network	96.03	93.15
Arabasadi et al. [66]	Hybrid neural network-genetic algorithm	88	91
Mokeddem and Ahmed [47]	Fuzzy classification model	87.39	94.38
Bashir et al. [26]	Ensemble model	73.68	92.86
Leema et al. [67]	Differential Evolution + BPNN$^{2}$	82.35	92.31
Mokeddem and Atmani [68]	Decision Tree + Fuzzy Inference System	92.44	96.18

The values listed in the table represent the average performance on ten folds

$^{1}$ Probabilistic principal component analysis

$^{2}$ Back propagation neural networks

**Table 19:** Comparison of the proposed system outcome with previous researches for Hungarian dataset

Author	Method	Recall (%)	Specificity (%)
Present study	Ensemble classifier	82.39	92.02
Shah et al. [64]	PPCA + SVM	80.43	88.42
Arabasadi et al. [66]	Hybrid neural network-genetic algorithm	85	88
Mokeddem and Ahmed [47]	Fuzzy classification model	82.98	90.57
Mokeddem and Atmani [68]	Decision Tree + Fuzzy Inference System	90.42	79.24

The values listed in the table represent the average performance on ten folds

## Discussion

Nowadays, numerous classification methods have been utilized for heart disease diagnosis. However, most of them concentrate on maximum the classification accuracy without taking the unequal misclassification costs into consideration. Therefore, the aim of this study is to propose a new ensemble method to tackle the deficiency of previous studies and improve the classification accuracy and reduce the misclassification cost simultaneously. The main contributions of the proposed research are as follows:

1. The proposed ensemble is a novel combination of heterogeneous classifiers which had outstanding performance in previous studies [15–19]. The limitations of a certain classifier are remedied by other classifiers in this model, which improves its performance.
2. We have used a new index to combine the results of individual classifiers. The proposed ensemble model not only focuses on high classification accuracy, but also concerns the costs patients have to pay for misclassification.
3. Compared with five individual classifiers and previous studies, the proposed ensemble classifier has achieved excellent classification results. The ensemble classifier gained significantly better performance than individual classifiers on all three heart disease datasets.

Kononenko [53] applied various machine learning techniques and compared the performance on eight medical datasets using five different parameters: performance, transparency, explanation, reduction, and missing data handling. While individual classifiers have shortcomings on some of these aspects, the proposed ensemble is able to overcome their deficiencies. For example, RF can generate explicit rules for decision making, and the basic idea of KNN is “to solve new problems by identifying and reusing previous similar cases based on the heuristic principle that similar problems have a high likelihood of having similar solutions” [54], which is easily understood by physicians. On the other hand, LR, SVM and ELM are more like a “black box”, and physicians are willing to accept a “black box” classifier only when it outperforms a very large margin all other classifiers, including the physicians themselves, but such situation is highly improbable [53]. In addition, KNN is a lazy evaluation method while the other four are eager evaluation methods. Eager algorithm generates frequent itemset rules from a given data set and predicts a class for test instance based on multicriteria approach from selected frequent itemset rules [26]. If no matching is found, default prediction (i.e., the most frequent class in data set) is assigned, which may not be correct. In contrast, lazy algorithm uses a richer hypothesis space, it makes judgment according to a small proportion of the instances in the database, thus overcomes the limitation of eager algorithms. However, lazy algorithm uses more time for prediction, as multicriteria matching is performed for each instance in data set [55], while eager algorithm is able to generate the prediction results at a very fast speed after the training phase. From the above discussion, it can be concluded that the selected classifiers complement each other very well. In any scenario where one classifier has some limitations, the other classifier overcome them. As a result, better performance is achieved. For this reason, we have used a combination of both lazy and eager classification algorithms.

Moreover, the present study takes MC into consideration and tries to reduce it. Most traditional algorithms focus only on the classification accuracy, ignoring the cost patients have to pay for misclassification. But the diagnostic mistakes are of higher importance in the medical field, and the price of a false negative instance is clearly much higher than that of a false positive one. Aiming at this problem, the present study has adopted a new method to combine the prediction results of heterogeneous classifiers and significantly reduced the MC, which could relieve patients from suffering.

Overall, the proposed model has following advantages compared with the state-of-the-art methods [56–59] :

1. The proposed ensemble outperforms the individual and ensemble classifiers in all three data sets which contain different feature spaces, which means that its generalization ability is outstanding. In contrast, most previous studies used only one data set [17, 18, 25], and that weakened the persuasive power of their results.
2. As the cost associated with missing a patient (false negative) is clearly much higher than that of mislabeling a healthy one (false positive), considering different kinds of misclassification cost makes the proposed method closer to reality.
3. This paper combines accuracy and MC as one evaluation metric, so the ensemble classifier is able to improve the accuracy and reduce MC at the same time. However, there are also shortages and limitations:
  1. The experiment did not take training time into consideration. The ensemble classifier needs longer training time than individual classifiers.
  2. The proposed approach doesn’t include state-of-the-art techniques such as deep neural network and soft computing method, which would be beneficial in improving its performance.
  On the whole, we believe that the proposed ensemble can be a useful tool in aiding physicians in making better decisions.

## Conclusions

In this study, a cost-sensitive ensemble method based on five different classifiers is presented to assist the diagnosis of heart disease. The proposed study takes full account of unequal misclassification cost of heart disease diagnosis, and employs a new index to combine various classifiers. In order to verify the performance of our proposed approach, the ensemble classifier was tested on Statlog heart disease dataset, Cleveland heart disease dataset and Hungarian heart disease dataset. Then, it was evaluated by different parameters such as E, MC, G-mean, precision, recall, specificity and AUC. Relief algorithm was utilized to select the most important features and eliminate the effect of irrelevant features. The significance of the results were tested by Wilcoxon signed-rank test. The results demonstrated that the proposed approach could yield promising results for heart disease diagnosis in comparison to individual classifiers and some previous works. In the future, the time complexity of the proposed ensemble method will be investigated and optimized, and new algorithms can be incorporated into the ensemble classifier to improve its performance.

## Data Availability

The data used in this study is available in UCI Machine Learning Repository.

## Abbreviations

ESC: European Society of Cardiology
CA: Coronary angiography
ECG: Electrocardiography
RF: Random forest
LR: Logistic regression
GA: Genetic algorithm
SVM: Support vector machine
ELM: Extreme learning machine
KNN: K-nearest neighbor
FCNN: Fully convolutional neural network
ACDC: Automated Cardiac Diagnosis Challenge
SLFNN: Single layer feed-forward neural network
MC: Misclassification cost
TP: True positive
TN: True negative
FP: False positive
FN: False negative
AUC: Area under curve
PSO: Particle swarm optimization
PPCA: Probabilistic principal component analysis
BPNN: Back propagation neural networks
